# 

**Published:** 1903-12-26

**Authors:** 


					/ the hospital. "The standard of highest purity."?Lancet. December 26th, 1903.
7 CADBURY'S
COCOA
"A PERFECT FOOD.
No. 900. Yol. XXXY. [alSfpen] Saturday, Dec. 26th, 1903. [SllbsCseeptp.?22lerms'] PRICE THREEPENCE.

				

